# Cerebral radiation necrosis successfully treated with high-dose bevacizumab

**DOI:** 10.1016/j.rmcr.2025.102282

**Published:** 2025-09-16

**Authors:** Keiichi Kugimiya, Hironobu Tsubouchi, Kiyotaka Saito, Yoshihito Kadota, Minako Azuma, Katsuya Sakai, Yasuharu Oda, Makoto Sumiyoshi, Shigehisa Yanagi, Taiga Miyazaki

**Affiliations:** a Division of Respirology, Rheumatology, Infectious Diseases, and Neurology, Department of Internal Medicine, Faculty of Medicine, University of Miyazaki, Kiyotake, Miyazaki, Japan; b Division of Neurosurgery, Department of Clinical Neuroscience, Faculty of Medicine, University of Miyazaki, Kiyotake, Miyazaki, Japan; c Department of Radiology, Faculty of Medicine, University of Miyazaki, Kiyotake, Miyazaki, Japan

**Keywords:** Cerebral radiation necrosis, Bevacizumab, Stereotactic radiosurgery

## Abstract

Cerebral radiation necrosis (CRN) is a late complication that can occur after the treatment of a brain tumor with focal radiation therapy, particularly stereotactic radiosurgery (SRS). Since an excessive production of vascular endothelial growth factor (VEGF) from necrotic lesions is a possible etiology of radiation necrosis, the anti-VEGF antibody bevacizumab has been reported as an effective treatment option. We report a case of a 71-year-old Japanese male with CRN following SRS, successfully treated with bevacizumab. He had presented with aphasia and right lower-limb muscle weakness 6 years after a left upper lobectomy for lung adenocarcinoma. Head magnetic resonance imaging (MRI) showed a metastatic brain tumor in the left temporal lobe. A craniotomy and pre- and post-operative SRS treatments were performed to relieve his neurological symptoms. Although initial symptom improvement was observed, the patient developed lower-limb muscle weakness and aphasia symptoms 7 months after the last SRS treatment. ^11^C-methionine positron emission tomography (PET) and ^18^F-fluorodeoxyglucose PET scans showed no abnormal uptake, leading to a diagnosis of CRN. The patient was treated with bevacizumab 15 mg/kg every 3 weeks for six cycles. The bevacizumab treatment resulted in an improvement of neurological symptoms and lesions showing gadolinium-enhancing effects and high-signal areas on T2-weighted fluid attenuated inversion recovery on MRI. The improvement was maintained 44 months after the completion of the last bevacizumab treatment. Although no definitive number of cycles and dosage of bevacizumab for CRN have been established, this case suggests that administering six cycles of bevacizumab may prevent long-term recurrence of CRN.

## 1 Introduction

Cerebral radiation necrosis is a late complication of radiotherapy that occurs in approximately 5 %–10 % of patients who have undergone radiotherapy [[1], [2], [3]]. The treatment strategy for symptomatic cerebral radiation necrosis (CRN) aims to manage symptoms and reduce the extent of necrosis [4,5]. Differentiating brain necrosis from brain metastases is often problematic due to their similar radiographic features, including lesion-associated edema and peripheral enhancement on T1-weighted post-gadolinium (Gd) magnetic resonance imaging (MRI) sequences [6,7]. However, noninvasive differentiation is important to determine the appropriate treatment strategy, as tumor progression may require surgical resection to improve survival. The ability to accurately differentiate between brain necrosis and brain metastases will contribute to optimal patient management and treatment outcomes.

The underlying cause of radiation-induced necrosis is thought to be a radiation-inducing upregulation of hypoxia inducible factor (HIF)-1α due to damaged oligodendrocytes and the subsequent release of vascular endothelial growth factor (VEGF) [1]. The released VEGF induces increased vascular permeability, angiogenesis, and subsequently perilesional edema [8]. Due to their anti-inflammatory properties, corticosteroids (particularly dexamethasone) are used as the first line of treatment for symptomatic cerebral radiation necrosis [5]. Corticosteroids effectively reduce perilesional edema and alleviate neurological symptoms, but they provide only symptomatic relief from radiation necrosis without addressing the underlying pathology, such as an increased induction of VEGF from the perinecrotic area of necrotic cerebral foci, which is the change in the central perilesion environment that causes edema. According to a systematic review, corticosteroid-refractory radiation necrosis remains a significant clinical challenge that necessitates alternative therapeutic strategies [5]. Unfortunately, prolonged corticosteroid use is associated with numerous adverse effects, including immunosuppression, exacerbation of metabolic disorders, and muscle weakness [4].

Since the pathophysiology of radiation necrosis is centered on the overexpression of VEGF [9], the monoclonal anti-VEGF antibody bevacizumab has emerged as a novel therapeutic option for cerebral radiation necrosis [10]. Randomized controlled trials have shown that bevacizumab treatment contributes to the reduction of necrotic lesions and the improvement of neurological symptoms [11,12]. However, the optimal dose for the treating CRN has not been established, and there are few reports on the long-term efficacy of this treatment.

Here, we report the case details of a patient with CRN caused by stereotactic radiotherapy (SRT) who was successfully treated with bevacizumab and did not experience recurrence four years after completing treatment.

## 2 Clinical report

**Fig. 1:**
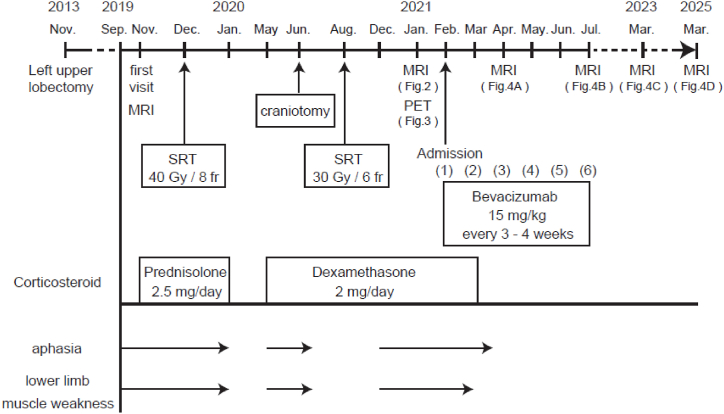
The patient's clinical course. The numbers in brackets refer to the bevacizumab treatment cycles. SRT: stereotactic radiosurgery.

In September 2019, a 71-year-old Japanese man presented with conduction aphasia, phonological aphasia, and right lower limb muscle weakness 6 years after having undergone a left upper lobectomy for lung adenocarcinoma (Fig. 1). An MRI scan revealed a metastatic brain tumor in the left temporal lobe, measuring 38.8 mm × 43.6 mm x 35.1 mm at 2 months after the onset of symptoms. SRT was initially performed with a total dose of 40 Gy (Gy) in eight fractions, followed by corticosteroid treatment to reduce brain edema in December 2019. At 6 months after the stereotactic radiosurgery a craniotomy was conducted to remove the temporal tumor lesion, due to insufficient symptomatic improvement. To prevent local recurrence, additional SRT was conducted to the surgical margins, where MRI showed Gd enhancement in August 2020. Following the craniotomy and additional radiotherapy, corticosteroid treatment was continued and the patient did not exhibit any specific neurological symptoms such as aphasia or muscle weakness.

**Fig. 2:**
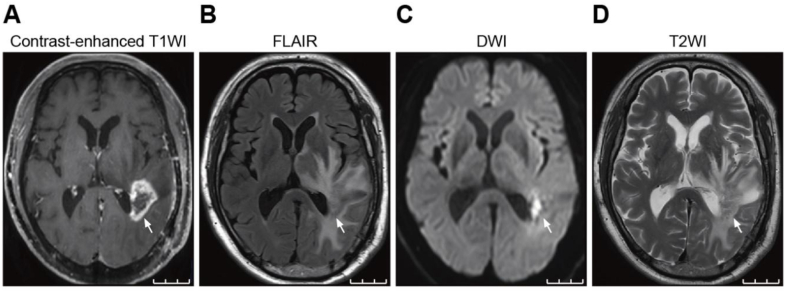
MRI of the brain lesions before treatment with bevacizumab. Contrast enhanced T1-weighted image (T1WI) **(A)**, T2-weighted fluid-attenuated inversion recovery (FLAIR) image **(B)**, and diffusion-weighted image (DWI) **(C)** acquired prior to bevacizumab treatment are shown. Contrast-enhanced T1WI shows an irregular enhancing lesion protruding into the excisional cavity in the left temporal lobe (arrows). The lesion exhibits high signal on DWI, low signal on the ADC map, and surrounding FLAIR hyperintensity. Scale bars: 30 mm.

**Fig. 3:**
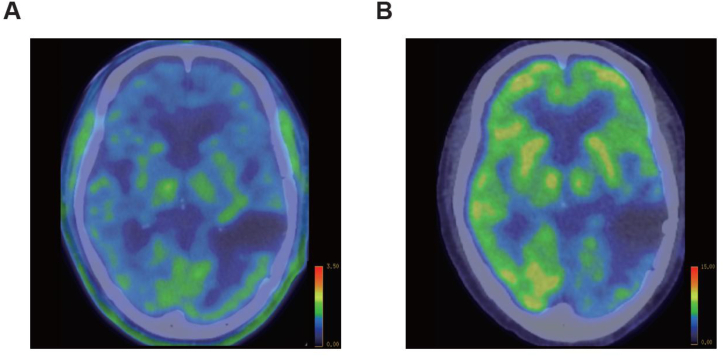
PET/CT fusion images. ^11^C-methionine PET image **(A)** and ^18^F-fluorodeoxyglucose PET image **(B)** at the patient's diagnosis of cerebral radiation necrosis (CRN). The standardized uptake value (SUV) is represented by the color scale.

Approximately 4 months after the additional stereotactic radiosurgery, aphasia, right lower limb weakness, and gait disturbance appeared. At the patient's admission to our institution, he was alert and oriented, and his vital signs were normal. His aphasia symptoms included word recall and phonological abnormalities. Chest computed tomography (CT) showed no metastasis in the lungs. A head MRI examination revealed irregular margins with enhancing effects protruding into the excisional cavity in the patient's left temporal lobe on contrast enhanced T1-weighted images (T1WI) (Fig. 2). The interior of the lesion showed high signal intensity on diffusion-weighted imaging (DWI) and low values on the apparent diffusion coefficient (ADC) map. T2-weighted fluid-attenuated inversion recovery (FLAIR) imaging demonstrated high signal intensity lesions surrounding the irradiated area. To differentiate between recurrent brain metastases of lung adenocarcinoma and radiation necrosis, methyl-^11^C-L-methionine (^11^C-MET) positron emission tomography (PET) examinations (Fig. 3A) and ^18^F-fluorodeoxyglucose (^18^F-FDG)-PET (Fig. 3B) were performed. The PET/CT fusion images revealed no abnormal accumulation of brain lesions.

**Fig. 4:**
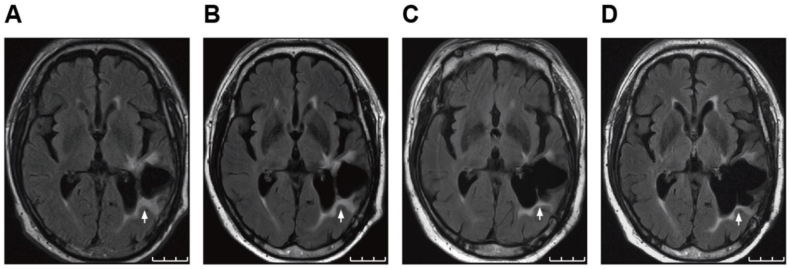
T2-weighted FLAIR MRI following bevacizumab treatment. Images were acquired after two cycles **(A)**, after six cycles **(B)**, at 20 months post-treatment **(C)**, and at 44 months post-treatment **(D)**. Arrows indicate areas of high signal intensity on T2-weighted FLAIR MRI, showing a gradual decrease during and after bevacizumab treatment.

Based on the absence of abnormal uptake on PET/CT fusion images, the patient was diagnosed with CRN. We treated the patient with bevacizumab 15 mg/kg every 3 weeks for six cycles. The MRI examination conducted after two cycles of bevacizumab treatment demonstrated a significant reduction in the surrounding edema and lesion size with irregular contrast enhancement (Fig. 4A). A further reduction in the size of the edematous lesion and the Gd-enhanced lesion were observed upon the patient's completion of the six treatment cycles (Fig. 4B). The patient then showed notable improvements in his aphasia symptoms (such as word recall impairment) and muscle weakness, leading to independent living with no adverse effects from the bevacizumab treatment. Throughout the 44 months following the completion of bevacizumab treatment, MRI confirmed maintained lesion reduction (Fig. 4C and D), and no new neurological symptoms had emerged.

## 3 Discussion

We have described the case of a patient with a metastatic brain tumor of lung adenocarcinoma with aphasia and muscle weakness due to CRN. The bevacizumab treatment improved his relapsed neurological symptoms (muscle weakness and aphasia), resulting in a reduction in both T1-weighted Gd enhancement and perilesional edema on FLAIR images.

Differentiating radiation necrosis from brain metastases is challenging due to the common imaging features such as mass formation, edema, and contrast enhancement on MRI, caused by the breakdown of the blood-brain barrier [13]. Advanced imaging modalities including perfusion-weighted MRI [14], proton magnetic resonance spectroscopy [15], and ^11^C-MET PET [[16], [17], [18], [19], [20]] have been applied to improve diagnostic accuracy and differentiate between these two conditions. With regard to the utility of ^11^C-MET PET in the differential diagnosis of brain radiation necrosis and brain metastasis, brain tumors, including brain metastases, exhibit an elevated uptake of ^11^C-MET due to an increased expression of L-amino acid transporters [21], distinguishing the tumors or metastases from normal brain tissue because of their low uptake [[16], [17], [18], [19], [20]]. The results of a meta-analysis demonstrated that the diagnostic accuracy of ^11^C-MET PET in differentiating brain radiation necrosis from brain tumors (including metastasis) is sufficient, with 0.870 sensitivity and 0.813 specificity [20].

In our patient, the region surrounding the metastatic tumor extraction cavity in the left temporal lobe, which exhibited contrast enhancement, required differentiation from brain metastasis. However, the reduced tracer uptake on ^11^C-MET PET suggested radiation necrosis rather than metastasis. The imaging evidence supported the decision to initiate bevacizumab therapy in this patient's case. His outcome highlights the utility of ^11^C-MET PET in providing crucial diagnostic insights, facilitating more accurate treatment decisions in complex clinical cases.

Regarding the association between the occurrence of brain radiation necrosis and the pathology of cancer, adenocarcinoma histology in non-small cell lung cancer (NSCLC) is associated with an increased rate of radiation necrosis [22]. That study indicated that patients with adenocarcinoma had a 12-month cumulative incidence of cerebral radiation necrosis at 5.8 %, which was significantly higher than the 3.9 % observed in squamous cell carcinoma cases [22]. In addition, the presence of epidermal growth factor receptor (EGFR) mutation does not affect the incidence of brain radiation necrosis [23], but anaplastic lymphoma kinase (ALK) rearrangements in NSCLC further amplifies the risk, with a 12-month cumulative incidence at 18 % compared to 4 %–8 % in other genetic profiles within lung cancer [24]. These differences underscore the importance of considering histological and genetic factors when assessing a patient's radiation necrosis risk and planning the treatment of gadolinium-enhancing lesions with surrounding edema.

The efficacy of bevacizumab for reducing perilesional edema, alleviating neurological symptoms, and improving the quality of life in affected patients has been described [10]. The rates of response to bevacizumab as a treatment for CRN were significantly higher than those for corticosteroid treatment, with response rates at 65.5 % and 31.5 %, respectively. In another study, the reduction in lesion size on T1-weighted images post-Gd enhancement was 25.5 % in the bevacizumab-treated group, compared to 5.0 % in the corticosteroid treatment group [12].

Our patient was treated with a high dose of bevacizumab (15 mg/kg every 3 weeks for six cycles), but no adverse events such as hypertension, proteinuria, or pulmonary hemorrhage were observed. He safely completed all six cycles of treatment, achieving a marked reduction in necrotic volume and edema on MRI. The dosing strategy has varied in the treatment of CRN, typically ranging from 1 to 15 mg/kg administered every 2–3 weeks, with a median of four cycles (range 1–27 cycles) [1]. Recently, the efficacy and safety of low-dose (5 mg/kg) and high-dose (10 mg/kg) bevacizumab have been compared: a study of 75 patients treated with either low-dose or high-dose bevacizumab revealed that both dosages significantly reduced the radiation necrosis volume on MRI at 3 and 6 months, with no substantial difference in effectiveness between the two groups [25]. However, the incidence of grade ≥3 adverse events (including hypertension, hemorrhage, epilepsy, and venous thrombosis) was higher in the high-dose group (9.8 %) compared to the low-dose group (0 %), indicating that low-dose bevacizumab is equally effective and is associated with fewer severe side effects compared to high-dose bevacizumab.

Zhuang et al. reported that among 13 patients who responded to low-dose bevacizumab (5 mg/kg) administered for at least three cycles, 10 patients (76.9 %) experienced a relapse during a mean follow-up period of 10 months after the discontinuation of bevacizumab [26]. Of the 10 patients who relapsed, five were re-treated with bevacizumab and three responded positively to re-treatment. It was suggested that after bevacizumab treatment is discontinued, the expression of HIF-1α might increase again in the tissue surrounding the necrosis, which increases the vascular permeability and eventually leads to a recurrence of brain necrosis [9]. In the present patient's case, neurological improvement and a reduction in edema observed via imaging were maintained for up to 44 months post-treatment. Our literature search identified no comprehensive examination of the relationship between bevacizumab dosing and recurrence rates at extended follow-up intervals. Further research is thus required to elucidate the association between relapse rates and bevacizumab dosage.

In summary, we report a case of a patient with lung adenocarcinoma and cerebral radiation necrosis who had improvement in neurological symptoms, particularly muscle weakness and aphasia, after treatment with bevacizumab. Reports on the long-term outcomes of brain necrosis lesions after bevacizumab treatment remain limited. In this case, cerebral edema was successfully controlled without the use of steroids for 44 months after the final dose of bevacizumab. Bevacizumab may represent a valid treatment option for patients with symptomatic brain necrosis, especially in cases where the long-term side effects of corticosteroids are a concern.

## CRediT authorship contribution statement

**Keiichi Kugimiya:** Writing – original draft. **Hironobu Tsubouchi:** Writing – review & editing, Writing – original draft, Visualization, Resources, Methodology, Investigation, Data curation, Conceptualization. **Kiyotaka Saito:** Visualization, Resources. **Yoshihito Kadota:** Visualization, Resources. **Minako Azuma:** Visualization, Supervision. **Katsuya Sakai:** Visualization. **Yasuharu Oda:** Data curation. **Makoto Sumiyoshi:** Data curation. **Shigehisa Yanagi:** Data curation. **Taiga Miyazaki:** Writing – review & editing, Supervision.

## Declaration of competing interest

The authors declare that they have no known competing financial interests or personal relationships that could have appeared to influence the work reported in this paper.
